# Retraction: Vector competence of biting midges and mosquitoes for Shuni virus

**DOI:** 10.1371/journal.pntd.0007274

**Published:** 2019-03-13

**Authors:** The PLOS Neglected Tropical Diseases Editors and Staff

The staff is retracting this publication as it is an uncorrected duplicate of pntd.0006993. The editors and staff of *PLOS Neglected Tropical Diseases* apologize for this error and the delay in its recognition.
